# The Role of Gut Microbiota on Insulin Resistance

**DOI:** 10.3390/nu5030829

**Published:** 2013-03-12

**Authors:** Andrea M. Caricilli, Mario J. A. Saad

**Affiliations:** 1 Department of Immunology, ICB IV, University of São Paulo, Av. Prof. Lineu Prestes, 2415, Cidade Universitária, São Paulo, SP, Brazil; E-Mail: caricilli@gmail.com; 2 Department of Internal Medicine, State University of Campinas, Rua Tessália Vieira de Camargo, 126, Cidade Universitária, Campinas, SP, Brazil

**Keywords:** obesity, insulin resistance, gut microbiota, type 2 diabetes

## Abstract

The development of obesity and insulin resistance has been extensively studied in the last decades, but the mechanisms underlying these alterations are still not completely understood. The gut microbiota has been identified as a potential contributor to metabolic diseases. It has been shown that obese individuals present different proportions of bacterial phyla compared with lean individuals, with an increase in Firmicutes and Actinobacteria and a decrease in Bacteroidetes. This alteration seems to interfere with intestinal permeability, increasing the absorption of lipopolysaccharide (LPS), which reaches circulation and initiates activation of Toll-like receptor (TLR) 4 and 2 and LPS receptor CD14, leading to increased activation of inflammatory pathways. With these activations, an impairment of the insulin signaling is observed, with decreased phosphorylation of the insulin receptor, insulin receptor substrate (IRS) and Akt, as well as increased inhibitory serine phosphorylation of IRS-1. Altered proportions of bacterial phyla have also been demonstrated to interfere with host’s biochemical pathways, increasing energy extraction and depot in adipose tissue. Therefore, understanding the mechanisms by which the alteration in the gut microbiota produces different signaling activations and phenotype changes may offer an interesting opportunity for the treatment of obesity and type 2 diabetes.

## 1. Introduction

The epidemics of obesity and type 2 diabetes mellitus in the past 20 years have led to numerous investigations concerning the mechanisms that are responsible for the development of these diseases. The general view is that insulin resistance is an early alteration of type 2 diabetes mellitus and obesity, and both diseases are strongly influenced by genetics and environment [1,2,3,4,5]. Moreover, studies in the past ten years have shown that low-grade inflammation has an important role in the molecular mechanism of insulin resistance in these diseases [6,7,8,9,10] and more recently (within the past five years) a new component that has both genetic and environmental factors is also being studied: the gut microbiota [11,12,13,14,15,16,17]. 

This way, a paradigm has been dismantled: microorganisms should no longer be associated with pathogenesis, since both bacteria and their eukaryote hosts benefit from their cooperative relationships [18]. In humans, there are at least 100 trillion microbial cells, collectively called microbiota, distributed in complex and site-specific communities. As the genome of these bacteria—the microbiome—contains hundreds of genes that do not exist in the human genome [19], we can consider our symbionts as an important extra organ. 

This complex community—bacteria, eukaryotes, viruses and Archeae—in its majority cannot be cultured. The reasons for this limitation are unknown growth requirements of the bacteria, selectivity of the media that are used, stress imposed by the cultivation procedures, necessity of strictly anoxic conditions, and the difficulties on simulating the interactions of bacteria with other microbes and host cells [20]. Thus, a new approach was introduced, culture-independent sequencing [21,22,23], which made detection of microbial genes and disease-associated patterns in our gut microbiota possible. The bacterial component of the microbiota has been intensively studied in the past few years, including high-investment studies such as the Human Microbiome Project [24,25] and MetaHIT [26]. 

Using this new approach made it possible to detect three dominating bacterial phyla in the human gastrointestinal tract: the gram-positive Firmicutes and Actinobacteria, and the gram-negative Bacteroidetes. Firmicutes is known as the largest bacterial phylum, comprehending 200 genera, which includes *Lactobacillus*, *Mycoplasma*, *Bacillus*, and *Clostridium*. In spite of Actinobacteria being also a dominant phylum, it is usually missed by RNA gene sequencing and can only be detected by fluorescent *in situ* hybridization [20,27].

Although gut microbiota has been described as relatively stable concerning its composition until old age [28,29,30,31], this temporal consistency considers that numerous variables are being held constant [32]. For example, dietary changes have been shown to have significant effects on the microbiota. Shifting mice to a high-fat, high-sugar “Western” diet, from a low-fat, plant polysaccharide-rich diet, changed the microbiota within 24 h [33]. Likewise, shifting from a high-fat/low-fiber diet caused notable changes in the gut microbiota within a day [34].

## 2. Gut Microbiota Composition

Recent studies have associated alterations in the gut microbiota with increased energy harvest and storage, and increased capacity of fermenting and absorbing otherwise undigested carbohydrates [27,35,36,37]. Moreover, the gut microbiota plays an important role in the development of the immune system and helps maintain the intestinal homeostasis [38]. For instance, they are essential for the emergence of T cell subsets and the differentiation of gut B cells into IgA-producing plasma cells [39,40,41,42]. 

The early establishment of the gut microbiota begins specially during birth, when babies are exposed to innumerous microbes from different environments, which colonize them promptly. Depending on the delivery mode, babies are colonized by microbes from their mother’s vagina or from the skin [43,44]. The gastrointestinal tract is colonized first of all by facultative aerobes, then by anaerobes. When the facultative anaerobic bacteria populations grow, they consume the oxygen and generate an anaerobic environment [45]. In the first week of life, these bacteria form a reducing environment, which favors the succession of strict anaerobes.

Supporting the view that the environment is extremely relevant for the composition of the gut microbiota and sometimes preponderant to the genetic predisposition [46], a study comparing dizygotic and monozygotic twins revealed that there is no significant difference in the degree of similarity in their gut microbiota [47]. Moreover, a study performing co-housing [48] has shown that it is possible to exacerbate a phenotype by exchanging different gut microbiota between mice that had been raised separately and put together in the same cage after a period of time. 

Another environmental factor that can profoundly change the microbiota profile is the use of antibiotics. Several studies have shown that treatment with antibiotics leads to major alterations in the gut microbiota composition [46,49,50,51,52]. After treatment with antibiotics, a reduced resistance to colonization is observed, permitting that foreign microbes grow and lead to permanent changes in the structure of the microbiota. This alteration can even cause diseases [32]. Although the altered taxa are different among individuals, some of them are not able to recover months after treatment and there is decrease in the bacterial diversity in most of the cases studied. 

A subtherapeutic antibiotic therapy, shown by Cho and colleagues [52], led to exposure of microbiota with increased metabolic activity that were able to extract a higher proportion of calories from dietary complex carbohydrates that were relatively indigestible in the control mice. This treatment also induced increase in short-chain fatty acids production, which are the metabolic product of this activity and may be delivered in increased quantities through the portal circulation of the liver, enabling enhanced lipogenesis. This study indicates the possibility that modulation of the infant gut microbiome by antibiotics could have long-term consequences affecting adiposity and bone development [52].

## 3. The Development of Obesity is Influenced by Gut Microbiota Composition

Obesity has also been correlated with reduced bacterial diversity, altered expression of bacterial genes, and metabolic pathways [36]. Obese individuals have a differential proportion of particular phyla in their gut microbiota: fewer Bacteroidetes and more Firmicutes, compared with lean controls [36,53]. Some studies, however, point that the decrease in Bacteroidetes is accompanied by an increase in Actinobacteria rather than Firmicutes [47]. We have shown that Toll-like Receptor (TLR)2 knockout (KO) mice present increased relative proportion of Firmicutes and, slightly, of Bacteroidetes. However, these mice presented increased weight gain after 12 weeks of age compared with their controls and were obese after 20 weeks [46], suggesting that different proportions of bacterial phyla may lead to alterations that culminate in obesity.

The Bacteroidetes bins were shown to be enriched in phosphotransferase systems involved in microbial processing of carbohydrates, whereas the Firmicutes bins were shown to be enriched in transport systems. Seventy-five percent of the obesity-enriched genes were from Actinobacteria and twenty-five percent from Firmicutes, while forty-two percent of lean enriched genes were from Bacteroidetes. Functional analyses suggest that many of them were involved in carbohydrate, lipid, and amino acid metabolism [47,54]. These findings suggest that a core gut microbiome exists as shared genes, with important alterations in metabolic functions rather than alterations simply in the relative abundance of bacterial taxa [47]. 

This shift in the relative abundance of phyla is associated with increased capacity for harvesting energy from food and with increased low-grade inflammation. The increased capacity to harvest energy from nutrients observed in mice with increased proportion of Firmicutes and decreased proportion of Bacteroidetes seems to be related to the presence of genes encoding enzymes that break down polysaccharides that cannot be digested by the host, consequently with increased production of more fermentation end-products, mainly short-chain fatty acids, and their conversion to triglycerides in the liver. Moreover, regulation of host genes that promote deposition of lipids in adipocytes has been related to altered gut microbiota composition. The microbiota suppresses the expression of fasting-induced adipose factor, Fiaf, a secreted lipoprotein lipase (LPL) inhibitor. By suppressing Fiaf, colonization enhances LPL activity, increasing storage of liver-derived triglycerides [55]. Turnbaugh *et al.* in a study with *ob/ob* mice found that reduced calorie content in the feces of *ob/*+ and +/+ littermates as compares with lean mice [27]. 

However, Backhed *et al.* [56] did not find statistical difference between germ-free and conventionalized mice concerning the energy content in their feces; this observation could be explained by other molecular mechanisms, as mentioned above. Another possible mechanism suggested by this group would be the increased activation of AMP-activated protein kinase (AMPK) in the skeletal muscle and liver, increasing fatty acid oxidation and glucose uptake in the muscle. Phosphorylated AMPK stimulates fatty acid oxidation in peripheral tissues by phosphorylating acetylCoA carboxylase (Acc). Phosphorylation of Acc leads to inhibition of its activity, decreasing malonylCoA levels, which releases carnitine:palmitoyl transferase-1 (Cpt1). This enzyme catalyzes the rate limiting step for entry of long-chain fatty acylCoA into mitochondria, therefore increasing fatty acid oxidation [57].

This way, results from experiments performed in germ-free and *Bacillus*-associated wild-type (WT) mice also support an active role of microbiota in modulating fat oxidation and accumulation, suggesting that, different from early beliefs, dietary fats alone might not be enough to cause overweight and obesity. It has been demonstrated that transplantation of gut microbiota from mice with obese phenotype to germ-free or *Bacillus*-monoassociated lean WT mice leads to increased body weight gain [27,46,58]. Turnbaugh and colleagues have shown that gut microbiota transplantation from mice with a mutation in the leptin gene—the *ob/ob* mice—to germ-free WT lean mice led to increase body weight gain [27]. Likewise, Vijay-Kumar and colleagues have shown that transplantation of gut microbiota from mice genetically deficient in Toll-like Receptor (TLR)5 to germ-free WT lean mice leads to weight gain and other signs of metabolic syndrome in the recipients [58]. However, in contrast with *ob/ob* mice, TLR5 deficient mice and WT littermate mice presented similar relative abundances of Firmicutes and Bacteroidetes. However, UniFrac analysis indicated that TLR5 deficient mice and WT littermate mice were different in their species composition. Moreover, the knockout mice presented increased or decreased phylotypes from various phyla. We have shown that transplantation of gut microbiota from mice genetically deficient in TLR2 to *Bacillus*-monoassociated WT lean mice leads to weight gain, insulin resistance and impaired insulin signaling, resembling the phenotype found in TLR2 deficient mice [46]. These metabolic characteristics were also similar to what was found in TLR5 deficient mice, except for the fact that TLR2 knockout mice did not present hyperphagia. However, regarding the gut microbiota composition, TLR2 knockout mice were different from TLR5 knockout mice, presenting increased relative abundance of Firmicutes compared with WT mice.

**Figure 1:**
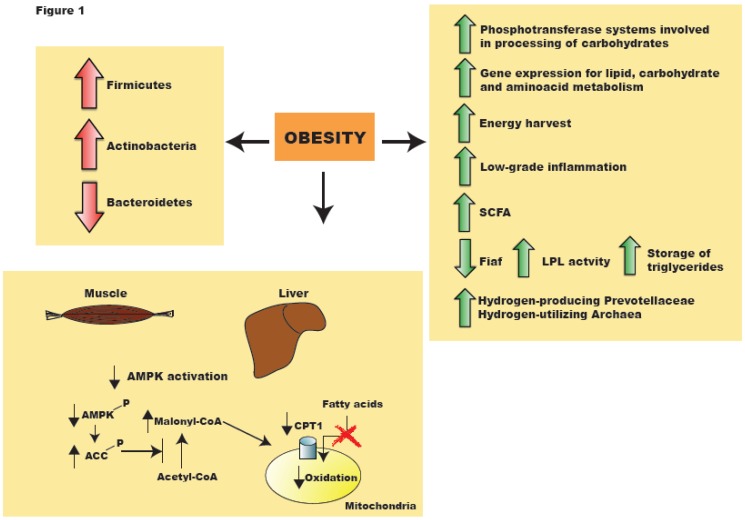
Alteration in gut microbiota composition due to obesity is accompanied by changes in activation of enzymes and pathways, leading to and increased inflammatory state and energy harvest. AMPK: AMP-activated kinase; SCFA: short-chain fatty acids; LPL: lipoprotein lipase; ACC: acetyl-CoA carboxylase; CPT1: carnitine palmitoyltransferase I.

It has also been suggested that obese individuals might be able to extract more energy from nutrients due to hydrogen transfer between taxa, as a concurrent increase in both hydrogen-producing Prevotellaceae and hydrogen-utilizing methanogenic Archaea has been associated with obesity [59]. Another study corroborates these data, showing that germ-free mice colonized with *Bacteroides thetaiotaomicron*, a saccharolytic member of the normal human colonic microbiota, together with the dominant human colonic methanogen, *Methanobrevibacter smithii*, have increased polysaccharide fermentation, increased de novo lipogenesis and enhanced host adiposity compared with animals with either organism alone [35] (Figure 1).

## 4. Gut Microbiota in Type 2 Diabetic Individuals

Recently, research has pointed out that the intestinal microbiome might be an important contributor for the development of type 2 diabetes (T2D) [60]. The use of genome-wide association studies (GWAS) has achieved many elucidations in this matter [61,62]. Qin *et al.* characterized the gut microbiota of T2D patients and observed increase in membrane transport of sugars, branched-chain aminoacids transport, methane metabolism, xenobiotics degradation, and sulphate reduction. However, they observed decrease in the levels of butyrate biosynthesis, bacterial chemotaxis, flagellar assembly, vitamins and cofactors metabolism. This study has also shown that the gut environment of T2D individuals is one that stimulates bacterial defense mechanisms against oxidative stress and against drugs [63].

Changes in the composition of the gut microbiota of obese individuals also implicate changes in the concentration of short-chain fatty acids in their feces [64]. Of the SCFA produced during from microbial fermentation process, butyrate has an important role as an energy substrate for cellular metabolism in the colonic epithelium, while acetate and propionate are taken up by the liver as substrates for lipogenesis and glucogeneogenesis. These SCFA, especially butyrate, also contribute to modulation of gene expression in mammalian colonic epithelial cells. As butyrate acts as histone deacetylase inhibitor, it may regulate around 2% of the mammalian transcriptome [65]. For example, glucagon-like peptide (GLP)-1, a hormone with antidiabetic effects secreted by L-cells of the distal small intestine and colon, may have its secretion modulated by SCFA [66,67]. 

Therefore, diabetic individuals might also have differential secretion of hormones due to fermentation products of their altered gut microbiota.

## 5. The Association between Insulin Resistance and Gut Microbiota

Insulin is an essential hormone for the regulation of glucose homeostasis and initiates its biological effects through activation of the insulin receptor (IR), which occurs by autophosphorylation of IR [68], leading to tyrosine phosphorylation of several substrates such as the insulin receptor substrate (IRS)-1 and -2 [69]. After tyrosine phosphorylation, IRS-1 and IRS-2 bind and activate phosphatidylinositol 3-kinase (PI3-K) [69,70], which increases serine phosphorylation of Akt, leading to glucose transport in muscle and adipose tissue, glycogen synthesis in muscle and liver and lipogenesis in adipose tissue. The appropriate signaling of this pathway may be disrupted by several mechanisms. Some of them are: serine phosphorylation of IRS proteins by protein kinases such as c-Jun *N*-terminal kinase (JNK) and inhibitory κB kinase (IKK)-β, and decreased tyrosine phosphorylation of IRS-1 [71,72,73,74,75,76].

One of the important mechanisms by which JNK pathway can be activated is by endoplasmic reticulum stress activation. In both adipose tissue and liver of mice chronically fed with a high-fat diet, PERK and IRE1α phosphorylation and JNK activity are significantly increased compared with lean animals [77].

The insulin signaling may also be impaired by altered secretion of cytokines and chemokines. For example, in type 2 diabetic patients, circulating T cells produce higher levels of IL-17 and IFN-γ, leading to a pro-inflammatory state [78]. Other cytokines, such as tumor necrosis factor (TNF)-α and interleukine (IL)-6, have also been related to insulin resistance. TNF-α protein is elevated is adipose tissue from four different rodent models of obesity and diabetes and the inhibition of its expression leads to increased peripheral uptake of glucose. Likewise, modulation of TNF-α release from the cell surface by alteration in the expression of TNF-α converting enzyme (TACE), a disintegrin and metalloproteinase, or of tissue inhibitor of matrix metalloproteinase 3 (TIMP3) leads to glucose intolerance and vascular inflammation or to hypermetabolic lean phenotype [79,80,81]. IL-6 can also affect insulin signaling: its plasma concentration is inversely proportional to insulin sensitivity, which is hypothesized by IL-6-induced suppressors of cytokine signaling (SOCSs) expression. SOCSs usually act suppressing the effect of cytokines on insulin transduction steps, such as IRS-1 phosphorylation, PI3K activation or PKB activation [82,83].

Similarly, the chemokine monocyte chemoattractant protein (MCP)-1, is associated with induction of insulin resistance and dedifferentiation of adipocytes. It is up-regulated by insulin resistance-inducing cytokines as IL-6 in adipocytes, representing a molecular connection between insulin resistance and obesity [84,85,86].

Moreover, insulin signaling can be affected by the activation of the innate immune system. Toll-like receptors (TLRs) play an important role in the activation of innate immune responses in mammals by recognizing conserved pathogen-associated molecular patterns [87,88,89]. There are at least 11 members of the TLR family in humans and 13 in mice [90]. TLRs play a crucial role in the recognition of invading pathogens and the activation of subsequent immune responses against them. Individual TLRs recognize distinct pathogen-associated molecular patterns. The TLR family harbors an extracellular leucine-rich repeat domain, as well as a cytoplasmic domain that is homologous to that of the interleukin-1 receptor (IL1R1). Upon stimulation, TLR recruits IL1R1-associated protein kinases via adaptor MYD88 and finally induces the activation of nuclear factor-κB and mitogen-activated protein kinases, as well as the expression of inflammatory cytokines [91,92,93].

**Figure 2:**
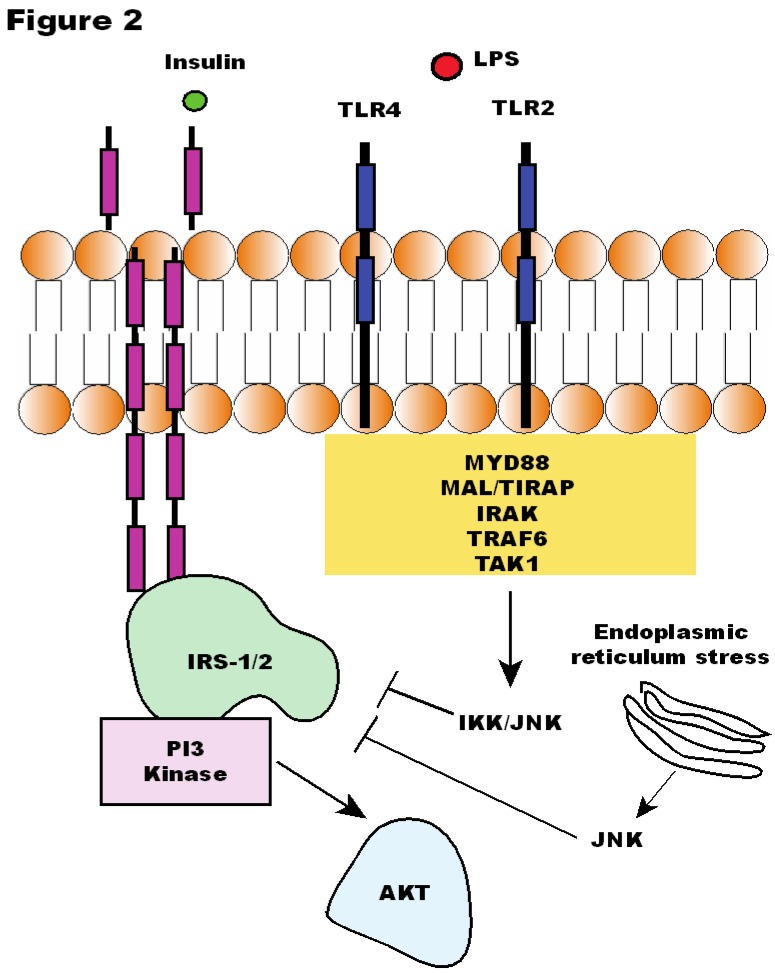
Insulin signaling is affected by Toll-like Receptor (TLR)2 and 4 signaling. LPS: lipopolysaccharide; IRS: insulin receptor substrate; PI3K: phosphoinositide 3-kinase; AKT: protein kinase B; IKK: IκB kinase; JNK: c-Jun *N*-terminal kinase; MyD88: Myeloid differentiation primary gene response (88); MAL/TIRAP: MyD88 adapter-like; TRAF6: TNFR-associated factor 6; IRAK: interleukin-1 receptor-associated kinase; TAK1: Transforming Growth Factor β-activated kinase 1.

TLR4 is a subclass of TLRs that can be activated by lipopolysaccharide (LPS), a major component of the outer membrane in Gram-negative bacteria, and by nonbacterial agonists, such as saturated fatty acids [73,94]. The activation of TLR4 signaling induces upregulation of inflammatory pathways related to the induction of insulin resistance, such as c-Jun NH2-terminal kinase (JNK) and IκB kinase complex (IKKβ)/inhibitor of nuclear factor-κB (IκBα)/nuclear factor-κB (NF-κB) [87,95], while loss-of-function mutation and knockout in TLR4 prevents insulin resistance induced by obesity or free fatty acids, suggesting an important role of TLR4 in the interface of innate immune system and energetic metabolism [95,96,97,98,99,100,101,102] (Figure 2). 

In adipocytes, two different mechanisms also contribute to LPS-induced insulin resistance. Activation of TLR4 by LPS in preadipocytes increases the expression of several cytokines, mainly TNF-α and IL-6, impairing the insulin signaling in adipocytes [103]. LPS can also promote expression of NF-κB and activation of MAPK pathway in adipocytes with several target genes [14]. Moreover, a study has suggested that LPS can promote the expression of iNOS [97,103], which is also known as capable of interfering with the insulin signaling [104]. The effect of nitric oxide on insulin action may be worsened by the increase of LPS-induced release of TNF-α and IL-6. Moreover, excessive production of nitric oxide worsens insulin resistance by increasing levels of circulating fatty acids, due to hampering LPL activity and increasing of lipolysis [105].

Likewise, TLR2 has been shown as an important modulator of insulin resistance. A recent study showed that palmitate treatment of differentiated C2C12 myotubes resulted in a time-dependent inhibition of insulin-activated signal transduction, through TLR2 activation [106]. We have also shown that short-term inhibition of TLR2 expression using TLR2 oligonucletide antisense in diet-induced obese mice leads to increased insulin sensitivity and signaling [107]. Other studies [108,109] have reported that TLR2 knockout (KO) mice present decreased body weight and adiposity, are protected against insulin resistance, and gain less weight on a HFD than control mice and are also protected against related comorbidities [55,110] (Figure 2). 

However, we have found an opposite metabolic description for TLR2 knockout mice. TLR2 KO mice in conventionalized conditions in our breeding center have insulin resistance and glucose intolerance associated with alterations in the composition of the gut microbiota, which displayed an increase in the relative abundance of Firmicutes and Bacteroidetes and decreased relative abundance of Proteobacteria, compared to their controls. The insulin resistance of TLR2 KO mice was accompanied by a down-modulation of insulin-induced insulin signaling in the liver, muscle, and adipose tissue, associated with an increase in endoplasmic reticulum stress. These metabolic alterations were characterized in eight-week-old TLR2 KO mice. At this age, knockout and WT mice had similar body weights. In spite of the fact that our knockout mice and those used in the other studies mentioned above had the same genetic background, they were bred in different rooms and fed with food from different sources, which can certainly have a role in the establishment and maintenance of gut microbiota [111]. This way, gut microbiota per se can subvert a genetically predetermined condition previously described as being protective towards obesity and insulin resistance into a phenotype associated with weight gain and its complications, such as glucose intolerance and diabetes [46].

**Figure 3:**
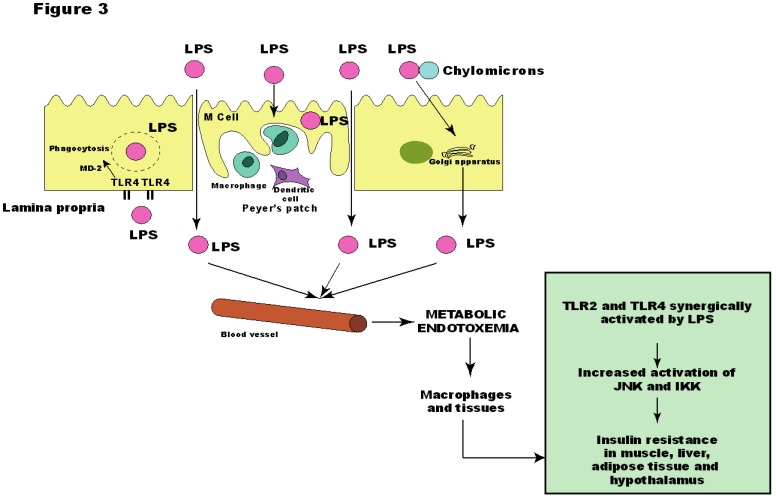
Metabolic endotoxemia leads to activation of insulin resistance in muscle, liver, adipose tissue and hypothalamus through activation of Toll-like receptor (TLR)2 and 4. JNK: c-Jun *N*-terminal kinase; IKK: IκB kinase; LPS: lipopolysaccharide; MD-2: myeloid differentiation factor-2.

Although the molecular origin of the state of low-grade inflammation found in obese individuals is unknown, LPS has been associated as the responsible one for the onset of metabolic diseases [97], since a continuous low-rate infusion of LPS induced most of the features of metabolic diseases, which did not occur in LPS receptor CD14 knockout mice [12]. Thus, we have found that the insulin resistance presented by TLR2 KO mice could be explained by the increased LPS serum levels, which led to increased activation of TLR4 in muscle, liver and adipose tissue, and increased phosphorylation of JNK, but not of IKK. Likewise, these mice presented reduced serum levels of proinflammatory citokynes IL-6 and TNF-α [46] (Figure 3).

The absence of increased IKK pathway activation in our model was very intriguing and can be explained by the cooperation between TLR4 and TLR2 signaling, as demonstrated by Laflamme and colleagues. This cooperation is evident when LPS is injected in TLR2 KO mice. After the first bolus of LPS, TLR2 KO mice show a robust signal for genes encoding innate immune proteins in the brain. However, the second LPS infusion failed to trigger TNF-α in TLR2 KO mice. These results indicate that TLR2 is involved in the second wave of TNF-α expression after LPS and that there is an elegant cooperation between TLR2 and TLR4 [112]. Therefore, it is possible that the development of metabolic diseases occurs through TLR2 and TLR4 after LPS stimuli, leading to a proinflammatory state and impairment of the insulin signaling.

High-fat feeding has also been correlated with increase in Gram-negative/Gram-positive ratio [12]. These findings also support the thought that intestinal microbiota could be responsible for changes in metabolic state, leading to endotoxemia and metabolic diseases. Treating high-fat diet-fed mice with antibiotics reduced normal plasma LPS values, reducing the occurrence of adipose tissue inflammation, oxidative stress and macrophage markers, as well as preventing adipocyte hypertrophy and improving metabolic parameters of diabetes and obesity in high-fat diet-fed mice [11]. 

This way, dietary fats can be associated with increased absorption of LPS, which might be related to changes in the gut microbiota, characterized by reduction in Gram-negative *Bacteroides*-like bacteria, in the *Eubacterium rectale-Clostridium coccoides* group, and in Bifidobacteria [113,114].

Although it is generally assumed that insoluble cereal fiber has beneficial effects that arise from chronic intake and its subsequent fermentation in the colon [114,115,116,117,118], the explanation for its consequent reduction of diabetes risk is still unclear. A study shows that in the first six weeks of high-cereal fiber (HCF) diet administration for overweight patients, an increase in the insulin sensitivity is observed. However, after 18 weeks, no significant differences are observed compared to control and high-protein diet [119]. Moreover, the HCF diet effects observed in whole body insulin sensitivity were not accompanied by changes in the composition of the gut microbiota neither after 6 weeks nor after 18 weeks of diet administration [120]. Therefore, other mechanisms might be responsible for these effects. After 18 weeks, prevention of aminoacid-induced activation of mTOR/S6K1 is one of the possible explanations for these results [119,120]. 

It is also relevant to state that most of the results with administration of diets or drugs are usually obtained from short-term studies, which hampers the transposition of conclusions to humans with dietary habits carried on for most of their lives. For instance, the metabolic effects of guar gum consumption can be completely opposite depending of the duration of its administration [121,122].

In addition, it is unclear whether results obtained from studies using animal models can be applies to humans since they present differences regarding the composition of gut microbiota and diet. 

## 6. Other Bacterial Factors may Contribute to Insulin Resistance

Other bacterial factors may play a role in the development of the insulin resistance. Like TLRs, nucleotide oligomerization domain (NOD)-1 and -2 proteins are intracellular pattern recognition receptors that sense bacterial cell wall peptidoglycan (PGN) moieties, which induce stress and inflammation pathways. NOD1 detects PGN structures found in gram-negative bacteria, whereas NOD2 detects PGN segments typically found in gram-positive strains [123]. Recent studies have associated NOD1- and NOD2-activating bacterial motifs with insulin resistance. Administering PGN-based NOD1 activator to adipocytes leads to activation of inflammatory programs, impairing insulin signaling and decreasing insulin-stimulated glucose uptake [124]. Likewise, PGN motifs that act on NOD2 induce muscle cell-autonomous insulin resistance [125]. NOD1-activating bacterial PGN motifs can also cause acute systemic insulin resistance in mice [126]. This NOD1 activation suppressed insulin action in the liver and in isolated hepatocytes, and decreased insulin-mediated glucose uptake in adipocytes [126]. Therefore, NOD1 ligand-mediated insulin resistance seems to involve crosstalk between cells from different tissues, likely adipose and hepatic, with indirect manifestation in skeletal muscle [123].

It is possible that many levels of regulation for NOD1-mediated sensing of PGN take place during obesity, since NOD1 transcripts were increased in epididymal adipose tissue of mice fed with high-fat diet [124]. It remains to be elucidated whether these NOD1 ligands, possibly derived from the gut microbiota, are altered during obesity and the extent of their contribution for the development of insulin resistance. 

Amar and colleagues have shown that after only one week of high-fat diet, bacterial translocation occurs towards adipose tissue and blood where inflammation is induced. This translocation is prevented in mice lacking the microbial recognition receptors NOD1 or CD14 [127], suggesting that these receptors have important roles in the development of the low-grade inflammatory state that characterizes insulin resistance.

Activation of TLR9 is another possible route by which bacterial components may contribute to development of other metabolic diseases. TLR9 recognizes bacteria-derived cytosine phosphate guanine (CpG)-containing DNA, activating innate immunity. Studies using TLR9 knockout mice have stated the relevance of this receptor in the development of non-alcoholic fatty liver disease, steatohepatitis and fibrosis. When its expression is ablated, suppression of IL-1β secretion and reduced steatohepatitis and fibrosis are observed. Together with TLR4, TLR9 leads to enhanced hepatic TNF-α expression and to the development of non-alcoholic steatohepatitis progression [128,129].

## 7. Intestinal Permeability and the Metabolic Endotoxemia

An important issue is how dietary fat increases LPS absorption. One explanation would be that LPS enters the body by transcellular transport through intestinal epithelial cells, which could occur through intestinal-epithelial microfold cells (M-cells). These cells are permeable to bacteria and macromolecules, and are responsible for helping sample gut antigens by the underlying lymphoid tissue [130]. 

It has also been reported that TLR is expressed on the apical surface of enterocytes, where it is capable of binding and internalizing purified endotoxin [131,132,133]. Neal and colleagues have demonstrated that enterocytes can internalize Gram-negative bacteria through TLR4, which mediates phagocytosis and translocation of Gram-negative bacteria in vivo [134]. LPS absorption might include internalization by enterocytes through myeloid differentiation protein-2 (MD-2)-dependent mechanism as well [134,135,136].

LPS can also be internalized by intestinal epithelial cells and transported to Golgi compartment of the enterocyte, where newly assembled chylomicrons are located before their basolateral secretion [137,138]. Recent reports have also suggested that diet may play an important role since LPS absorption from the gut was found to be associated with the ingestion of dietary fat [12,139]. Because the Golgi is a major compartment in chylomicron transport to the basolateral membrane [138,140,141] and because LPS has great affinity for chylomicrons [142], a study has raised the possibility that LPS could be associated with chylomicrons within the enterocyte [143], being LPS then secreted from cell-associated pools in a chylomicron-dependent manner.

Another possibility is that dietary fat leads to paracellular leakage of LPS across the intestinal epithelium. This is supported by the observation that intestinal tight-junction integrity is impaired in obese mice [144] and by studies in which intestinal luminal exposure to oleic acid can cause intestinal epithelial damage [145,146].

**Figure 4:**
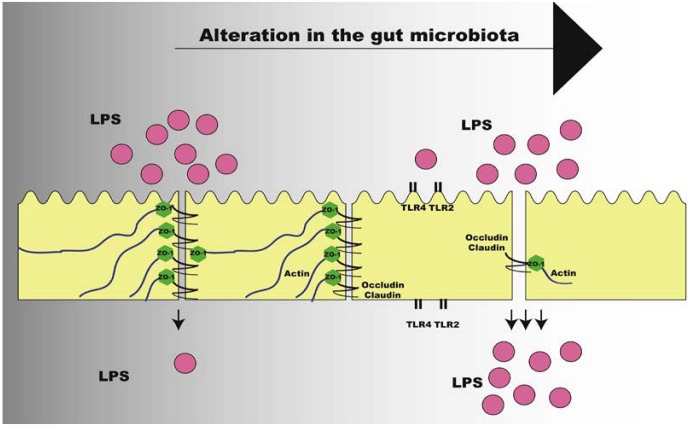
Alteration of intestinal permeability after change in gut microbiota of obese individuals. LPS: lipopolysaccharide; TLR: Toll-like Receptor; ZO-1: zonula occludens.

Studies showed that TLR2 regulates tight junction (TJ)-associated intestinal epithelial barrier integrity and that TLR2 deficiency predisposes to alterations of TJ-modulated barrier function leading to perpetuation of mucosal inflammation [147,148]. Following this direction, we have shown that TLR2 KO mice present increased LPS absorption, as demonstrated by LPS oral administration challenge, due to decreased expression of TJ protein zonula occludens (ZO)-1 in the ileum, which leads to increased gut permeability. Improvement of gut permeability has been associated with increase in *Bifidobacterium* spp., with higher expression of tight-junction proteins [149]. In our investigations, we have also observed that TLR2 KO mice presented decrease in *Bifidobacterium* spp., supporting the lower expression of ZO-1. After gut microbiota transplantation from TLR2 KO to *Bacillus*-monoassociated WT mice, we observed that a reduction in the expression of ZO-1 in the ileum occurred in the recipients, suggesting that the expression of this protein is regulated by the particular microbiota present in the gastrointestinal tract of TLR2 KO mice [46] (Figure 4). 

In the situation in which there is increased intestinal permeability and increased absorption of LPS, a state of metabolic endotoxemia is initiated, characterized by elevated serum LPS concentration, but still 10–50 times lower than values that could be reached in septicemia or other infections [12]. The origin of metabolic endotoxemia is still unclear, but it is strongly suggested that is may be associated with changes in the gut microbiota, leading to increased activation of inflammatory pathways and impairment of the insulin signaling.

## 8. Conclusions

Environmental alterations have been shown as important factors in the development of many diseases. Obesity and insulin resistance are long known for being mainly influenced by a positive balance between food intake and energy expenditure. In the past five years, a new component that has both genetic and environmental factors has also being associated with the development of obesity: the gut microbiota. It can be both altered by diet changes or drug exposition and by genetic factors. Likewise, it may also influence the expression of host’s proteins and enzymes, as well as its active biochemical pathways, directly or through their products of fermentation. These interactions are still poorly understood, although metagenomic tools have provided an enormous amount of data concerning the characterization of microbiota from different parts of the host’s body in different conditions. 

Changes in bacterial phyla proportions during obesity have captured science attention worldwide, especially because of their effects on metabolism. Increased proportion of Firmicutes and Actinobacteria and decreased proportion of Bacteroidetes have been associated with increased serum LPS levels, insulin resistance, increased body weight gain and other comorbities of the metabolic syndrome. The mechanisms that underlie this regulation are still unclear, but their unrevealing brings potential interventions for the treatment of obesity and type 2 diabetes. 
